# Global Dynamic Impression for Differentiating Between Epileptic and Psychogenic Nonepileptic Seizures: A Feasibility Study

**DOI:** 10.1002/brb3.70130

**Published:** 2025-01-20

**Authors:** Linor Avraham, Ronen Spierer, Raz Winer, Noam Bosak, Danielle Wasserman, Moshe Herskovitz

**Affiliations:** ^1^ Technion Faculty of Medicine Haifa Israel; ^2^ Neurology Department Rambam Health Care Campus Haifa Israel; ^3^ Neurology Department Shaare Zedek Medical Jerusalem Israel

**Keywords:** global dynamic impression, psychogenic nonepileptic seizures, survey

## Abstract

**Objective:**

Medical personnel show difficulty in differentiating psychogenic nonepileptic seizures (PNES) from epileptic seizures (ES). The purpose of this study was to conduct an initial feasibility assessment of the global dynamic impression (GDI) principle and to evaluate its effectiveness in enabling the diagnosis of epileptic versus psychogenic seizures using video footage of events, even by untrained personnel

**Methods:**

We based this study on video footage showing five videos of PNES and five ES videos. We asked physicians and nurses from the emergency department, internal medicine department, neurology department, and medical students to classify the videos before and after learning the GDI principle. The GDI principle is a simple clinical tool assuming repetitive movements with minimal dynamics in vector and frequency in motor PNES. A correct answer earned a score of 10 points, and a wrong answer a score of 0. Therefore, the questionnaire score could range from 0 to 100.

**Results:**

A total of 108 medical personnel participated in the study. A total of 42 participants filled out the questionnaire before training—Group 1, and 36 participants filled out the questionnaire after training—Group 2; 30 participants filled out the questionnaire before and after the training—Group 3. The mean score of Group 1 was 55.23 ± 17.83 versus 75.55 ± 13.61 in Group 2 (*p* = 0.0000001). The mean score of Group 3 was 53 ± 17.8 compared to 78 ± 12.9, before and after training, respectively (*p* = 0.000001).

**Significance:**

A brief training on the GDI principle of various medical teams, even unskilled teams, significantly improves differentiating PNES from ES.

## Graphical abstract

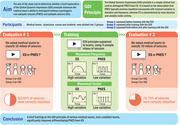

## 1 Background

In a previous study, we highlighted the significant challenges medical teams, particularly first responders, encounter when distinguishing between psychogenic nonepileptic seizures (PNES) and epileptic seizures (ES) (Wasserman and Herskovitz 2017). Although video EEG monitoring (VEM) is the gold standard for differentiating these seizures, its limited availability, hospitalization requirements, and necessity for a high seizure frequency can hinder its use (Herskovitz and Schiller 2017). The increasing prevalence of video footage from various environments underscores the importance of identifying semiological markers through video analysis. Tatum et al. (2021) evaluated outpatient smartphone videos and found that expert review accurately predicted video EEG diagnoses of ES 89.1% of the time, with enhanced accuracy when combined with patient history. Other studies have yielded similar findings (Amin et al. 2021; Karakas et al. 2021; Ramanujam, Dash, and Tripathi 2018; Ricci et al. 2021).

Birca et al. (2021) evaluated the ability of various healthcare professionals (HCPs) and students to distinguish between PNES and ES using video recordings. Following diagnostic accuracy reporting guidelines, 558 participants classified 36 seizure videos (18 PNES and 18 ES). Neurologists and epileptologists demonstrated the highest diagnostic accuracy, followed by neurology residents, other specialists, and undergraduate medical students. While trends suggested differences in accuracy among groups, statistical comparisons revealed no significant differences, and patient age and sex did not affect accuracy. Overall, recognizing PNES was correlated with the HCP's level of expertise in neurology and epilepsy.

Vinton et al. (2004) observed that during tonic–clonic seizures in epilepsy patients, muscle artifacts progressively accumulate and intensify during the tonic phase, then decrease and disappear during the clonic phase. In contrast, patients with PNES exhibit a sudden onset of muscle artifacts without the gradual buildup or decline, suggesting reduced motor dynamics. This observation led us to explore the application of these findings across all types of motor seizures, both in ES and in PNES (Winer, Shahkoohi, and Herskovitz 2024). To quantify the difference in motor dynamics between PNES and ES, we introduced the mean dynamic index (MDI), a scoring system designed to capture distinct motor patterns during motor‐predominant seizures. The body was divided into five regions—head and face, upper extremities, and lower extremities—excluding the trunk due to its limited movement potential. Only actively involved areas contributed to the score, with each distinct motor feature adding a point to the regional dynamic index (RDI). The MDI was calculated as the average of all RDIs. In this study of 15 patients with predominantly motor presentations, 8 were diagnosed with PNES, while 7 had ES.

The mean MDI was 1.2 ± 0.4 in the PNES group and 2.8 ± 0.77 in the ES group (*p* < 0.001). An MDI score of 1.665 provided 100% sensitivity and 87.5% specificity for distinguishing ES from PNES.

While the MDI showed promise as a bedside tool for differentiating ES from PNES, it presented challenges, particularly in recognizing novel unique motor patterns in ES, making it difficult for untrained personnel to apply consistently. In contrast, a holistic dynamic principle offered a more effective method, achieving complete differentiation between PNES and ES in this study of 15 cases. This principle eventually evolved into the global dynamic impression (GDI), which posits that PNES is characterized by fewer movement dynamics over time compared to ES.

The idea behind this approach was that, by presenting a small number of examples illustrating the contrast between high motor dynamics in ES and low motor dynamics in PNES, even untrained individuals could effectively distinguish between ES and PNES. Therefore, this prospective study aims to evaluate whether brief training in the GDI principle can enable untrained personnel to accurately differentiate between ES and PNES using video footage.

## 2 Methods

### 2.1 Video Recordings

Video footage was extracted from the first 10 consecutive patients (5 with PNES and 5 with ES) who underwent VEM at our center starting in November 2018, meeting the following criteria:

1. A definite diagnosis of either ES or PNES was made by a neurologist trained in epileptology, using clinical semiology and concurrent EEG.
2. Motor phenomena were observed in all selected cases.
3. For all selected ES, the video captured the beginning, evolution, and end of the seizure.
4. For PNES cases, due to the length of the seizures, only part of the event was shown, but with a distinct beginning and end.

All patients underwent anonymization; due to the fact that only medical teams viewed the films, we did not obscure the patients' faces

### 2.2 Observers

The current study is similar in design to our previous study (Wasserman and Herskovitz 2017), as most of the recruitment was done during morning meetings in the departments of Internal Medicine, emergency room, and neurology at Rambam Health Care Campus. Each participant recorded the last four digits of their ID number. Prior to participating, observers were informed about the study's purpose and assured that all information would remain anonymous. Each observer received a questionnaire that included questions about their professional background, medical position, years of experience, department, and their assessment of whether the observed event was an ES or a PNES.

In each department, we had two rounds of recruitment. In the first round, we explained about PNES and administered the test to everyone who was present at the meeting. At least two weeks later, we conducted a second round in which we explained about GDI and administered the test again. After the two rounds in each department, the participants were divided into three groups: Group 1: All those who were present only in the first round (observers before GDI training). Group 2: Those who were present only in the second round (observers after GDI training). Group 3: Those who were present in both rounds (observers who conducted the test both before and after GDI training). There was no overlap between the groups.

Since the epilepsy team conducted the study, no epileptologists were included in the neurology department observers.

### 2.3 Guidance and Training

As previously mentioned, Groups 1 and 3 performed the test before receiving GDI training. During this initial stage, we explained the nature of PNES and the importance of accurate diagnosis. Observers then watched the video events continuously, without the ability to pause or rewind.

Groups 2 and 3 completed the test after undergoing GDI training. For Group 3, which took the test both before and after the GDI training, we ensured a minimum two‐week interval between the sessions, as they viewed the same video footage in both tests. In addition, we took precautions to ensure that these observers did not receive any hints or feedback about the correct answers, to avoid improvement due to familiarity or repeated scoring of the videos.

### 2.4 GDI Principle

The GDI principle is rooted in a study conducted several years ago (Winer, Shahkoohi, and Herskovitz 2024). In that research, we developed a quantitative index, which we named “mean dynamic index” (MDI) to assess the average dynamism across all areas involved during a seizure. The study, carried out by two independent assessors (young and senior epileptologists) blinded to seizure types.

Although the dynamic principle successfully achieved a complete differentiation between PNES and ES in a series of 15 cases (8 PNES and 7 ES), the MDI was overly complex and unsuitable for clinical bedside use. Since our primary goal was to establish an effective method for instructing all healthcare practitioners encountering patients with PNES or ES on how to distinguish between these conditions, we realized that by examining the dynamic principle as a qualitative measure, we could attain a highly effective differentiation between the two.

**FIGURE 1:**
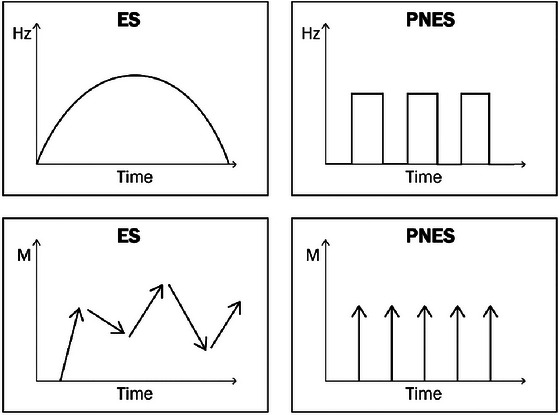
Global dynamic impression principle. The graph demonstrates the GDI principle, showing that the frequency and direction of ES change over time. In contrast, in PNES, both the frequency and direction of movement are fixed, appearing in an outbreak and disappearing suddenly.

The GDI principles assumes that the movements in motor PNES are repetitive with minimal dynamics in terms of movement vector and frequency. Main principle implies that regardless of the anatomical regions involved, in PNES, the movements in each anatomical region involved occurs in bursts, are constant, and repetitive—burst–arrest–burst—with similar movement patterns in each burst. Contrarily, during ES, the movements begin with a fading‐in phase and end with a fading‐out phase and are dynamic in terms of movement's vector and frequency during the events. Figure 1 summarizes the main principles of GDI. Given the significant challenge of explaining the GDI principle through words alone, we created an illustrative video (Video S1) to convey the concept more effectively. This video provided a detailed explanation of the GDI principle, as presented to the participants in our study. Observers were instructed to classify each video footage according to the GDI principle rather than to base them on their previous knowledge. GDI training took approximately 5 min. After training, the observers continuously saw the events without being able to stop in the middle or go back and forth on the video.

It is important to note that the absolute validity of the principle of dynamism is not yet established. The explanation provided to the participants is based on the hypothesis of eradicating dependency. The purpose of this initial research is to evaluate whether the principle effectively works.

### 2.5 Statistics

Statistical analyses were performed using SPSS v25 for Windows.

We treated the survey questionnaire as a test with multiple‐choice questions. Each question had two possible answers: PNES or ES. A correct answer to a question was awarded 10 points, so the test score ranged from 0 to 100.

For each group, a mean score was calculated. To compare the means between Groups 1 and 2, a one‐tailed nonpaired *t*‐test was used. Analysis of covariance (ANCOVA) was then conducted on scores between groups, with profession used as a covariate. For Group 3, comparing the means before and after GDI training, a one‐tailed paired *t*‐test was employed. Repeated measures ANCOVA (RM‐ANCOVA) model was used for Group 3, with profession as a between‐subjects factor. Two‐tailed nonpaired *t*‐tests were used to compare between the untrained participants (Groups 1 vs. 3—before), and between the trained ones (Groups 2 vs. 3—after). Statistical significance was defined as *α* < 0.05.

In addition, we calculated the sensitivity and specificity of the GDI principle for PNES, using the post‐training scores from participants in Groups 2 and 3. These calculations were performed with 95% confidence intervals (CIs).

### 2.6 Terminology

We are aware of the debate regarding the proper name for PNES (Asadi‐Pooya et al. 2020; Brigo et al. 2015).

Due to educational purposes we decide to use the term PNES in our study.

### 2.7 Ethics

The Institutional Helsinki Committee, application number RMB‐0446‐20, approved the study. The patients shown in Video S1 signed a consent form for the use of their recordings in this article.

## 3 Results

A total of 108 medical personnel participated in the study: 30 from the neurology department (16 nurses and 14 physicians), 18 from the emergency room (all physicians), 25 from internal medicine (5 nurses, 20 physicians), and 35 medical students. Table 1 summarizes the demography of all participations and participants in each group.

**TABLE 1:** Demographic of all participants.

Profession	All participants	Group 1: Before GDI training	Group 2: After GDI training	Group 3: Before and after GDI training
ER physicians	18	13	1	4
Internal medicine physicians	20	9	10	1
Internal medicine nurses	5	2	2	1
Neurology physicians	14	7	1	6
Neurology nurses	16	9	6	1
Medical students	35	2	16	17
Total	108	42	36	30

Groups 1, 2, and 3 included 42, 36, and 30 participants, respectively.

**FIGURE 2:**
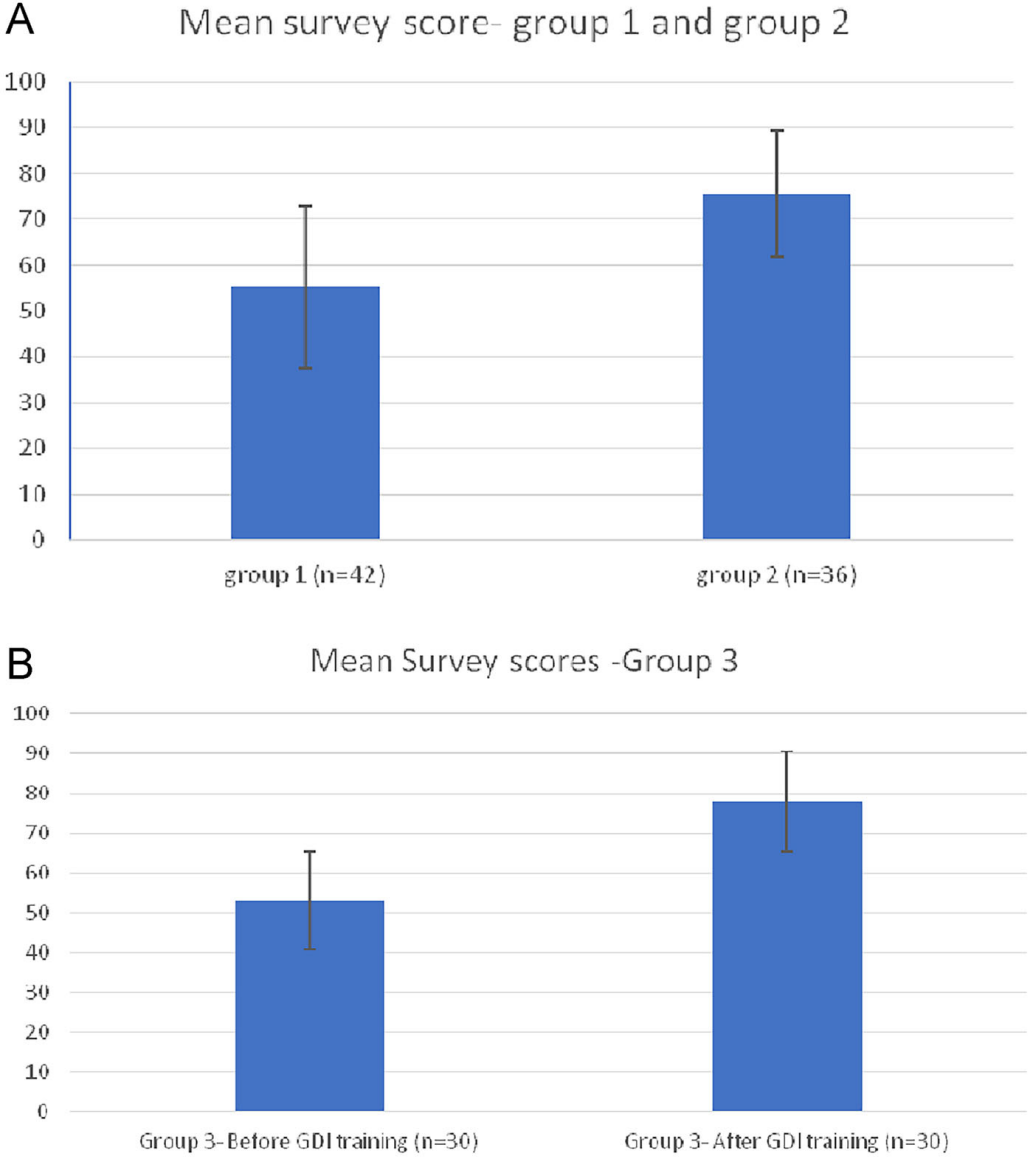
Survey results before and after GDI training.

The mean score of Group 1 was 55.23 ± 17.83 versus 75.55 ± 13.61 in Group 2 (*p* = 0.0000001). This difference persisted (*p* = 0.0001) in the ANCOVA. The mean score of Group 3 was 53 ± 17.8 compared to 78 ± 12.9, before and after training, respectively (*p* = 0.000001; Figure 2). This result persisted (*p* = 0.0001) in the RM‐ANCOVA. In each ANCOVA, there was no statistically significant difference between professions (*p* > 0.05).

**FIGURE 3:**
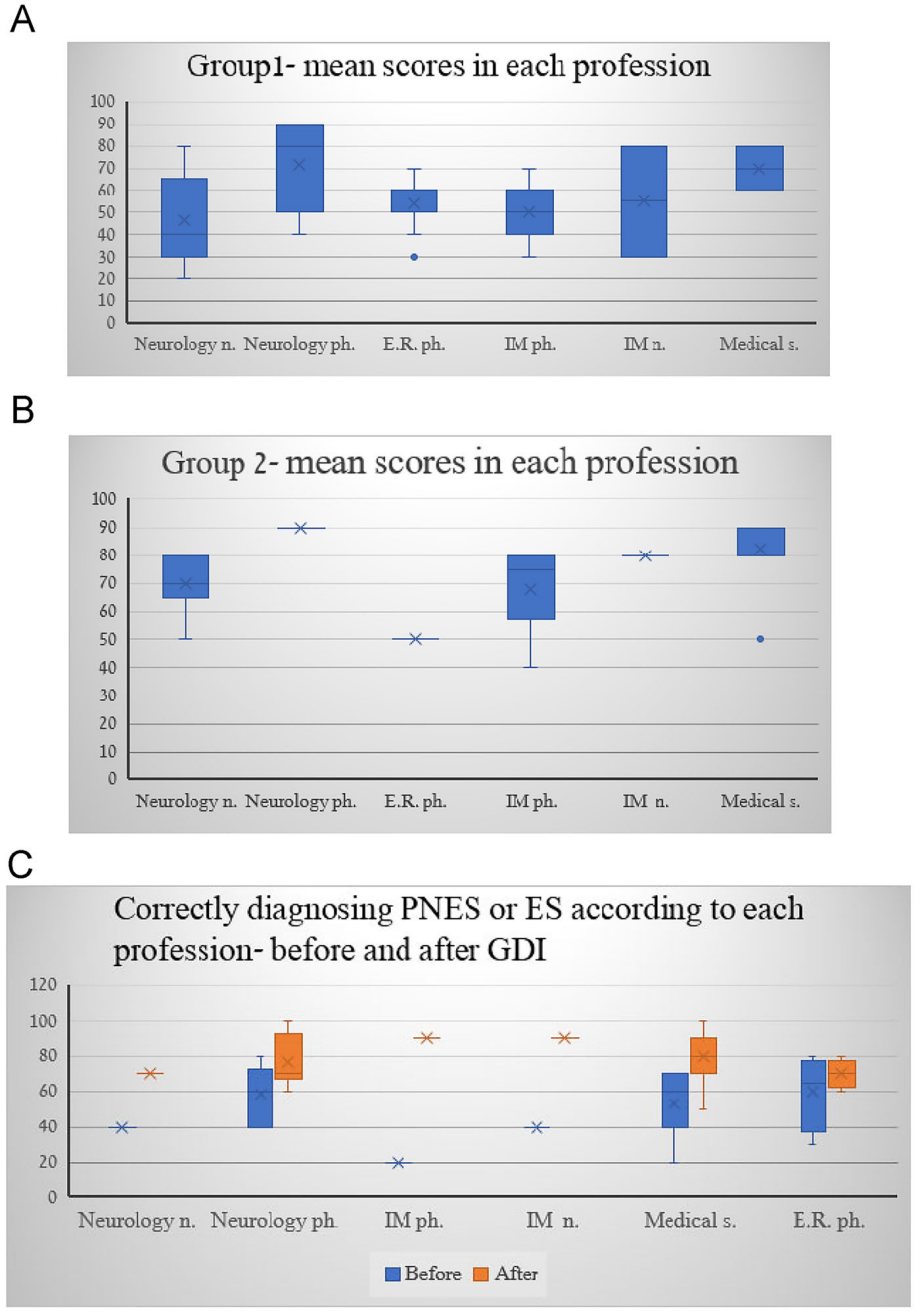
Mean scores in each profession for each group. E.R. ph., emergency medicine physician; IM n., internal medicine nurses; IM ph., internal medicine physician; Medical S., medical students; Neurology n., neuorlogy nurses; Neurology ph., neurology physician.

Figure 3A–C summarizes the scores of each group according to profession.

Neurology physicians in Group 1 scored significantly higher than all other observers 65.38 ± 18.9 (*p* < 0.05). In all the professional subgroups of Group 3, improvement was found after GDI training. The comparisons between the groups of untrained participants (1 vs. 3—before), and the groups of trained ones (Groups 2 vs. 3—after), were both insignificant (*p* > 0.05).

**FIGURE 4:**
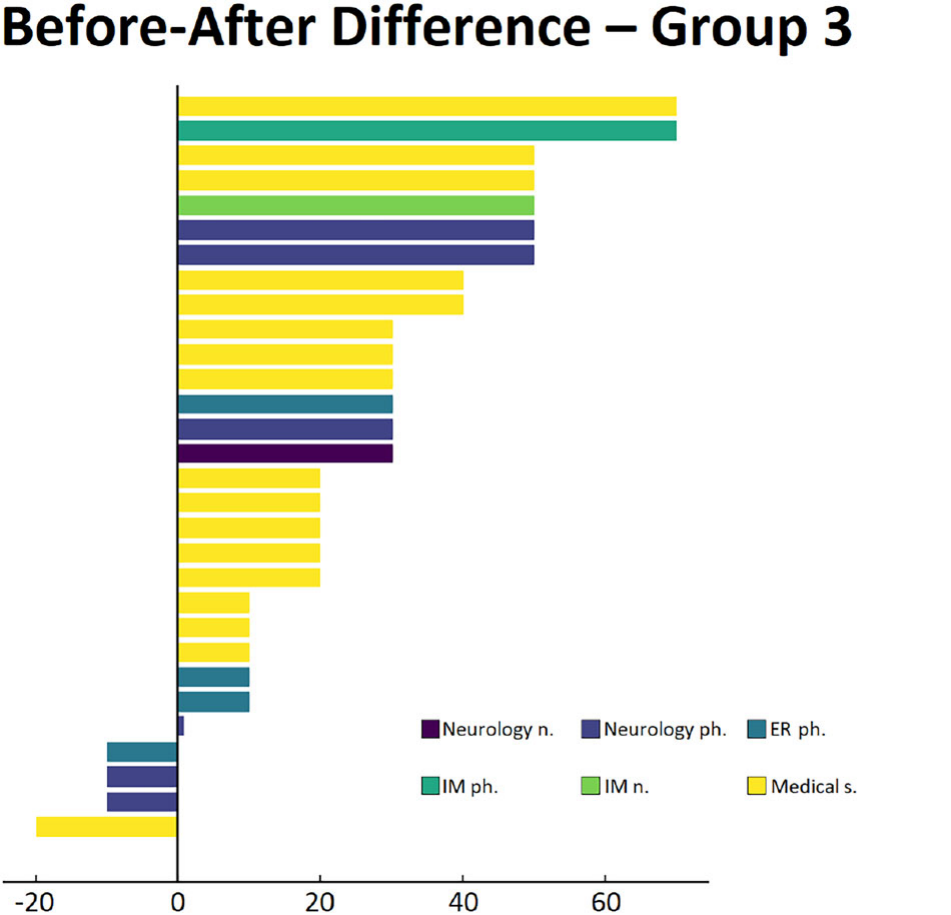
Before and after difference for all participants in Group 3. E.R. ph., emergency medicine physician; IM n., internal medicine nurses; IM ph., internal medicine physician; Medical S., Medical students; Neurology n., neuorlogy nureses; Neurology ph., Neurology physician.

Figure 4 summarizes the changes in scores for each participant in Group 3 before and after GDI training.

Table 2 summarizes the confusion matrix data for all participants before and after GDI training. The sensitivity and specificity of GDI principle in correct classification were 75.73% (95% CI: 70.83%–80.18%) and 77.67% (CI: 72.69%–82.13%), respectively.

**TABLE 2:** Confusion matrix: Demonstrating the ability to distinguish between PNES and ES in all participants, without or with the GDI principle.

Group	Time point	*N* ^a^	TP	TN	FP	FN	Specificity	Sensitivity	NPV	PPV
1	Before	42	119	119	88	94	0.57	0.56	0.56	0.57
3	Before	30	75	84	74	67	0.53	0.53	0.56	0.50
All	Before	**72**	**194**	**203**	**162**	**161**	**0.56**	**0.55**	**0.56**	**0.54**
2	After	36	134	138	46	42	0.75	0.76	0.77	0.74
3	After	30	125	109	25	41	0.81	0.75	0.73	0.83
All	After	**66**	**259**	**247**	**71**	**83**	**0.78**	**0.76**	**0.75**	**0.78**

^a^ Ten observations per participants.

## 4 Discussion

Differentiating between PNES and ES is challenging, especially for first responders, due to overlapping symptoms, leading to misdiagnosis (Wasserman and Herskovitz 2017). While VEM is the gold standard for diagnosis, its limited availability and need for hospitalization hinder its use (Herskovitz and Schiller 2017). This feasibility study aimed to check a new diagnostic tool, the GDI, which uses video analysis to distinguish between PNES and ES.

The increasing availability of video footage from public and private sources has provided a valuable resource for seizure analysis (Ricci et al. 2021). Studies show that smartphone home videos can reliably aid in diagnosing PNES and ES, with up to 97.2% concordance with video‐EEG (VEEG) and 95.4% sensitivity (Ramanujam, Dash, and Tripathi 2018). In 94% of cases, smartphone videos matched VEEG diagnoses, proving useful when VEEG is unavailable (Amin et al. 2021). A systematic review also highlighted that home videos improve diagnostic accuracy and reduce stress, though better video quality training is needed (Ricci et al. 2021). In veterans, home videos were 88% accurate in distinguishing seizures, with moderate inter‐rater reliability (Karakas et al. 2021). Video quality was sufficient in 70.8% of cases, but factors like short duration and limited interaction hindered accuracy (Tatum et al. 2021).

Video analysis enables the identification of semiological markers—observable features of seizures that can help distinguish between PNES and ES.

As mentioned earlier the ability to recognize PNES correlated with the HCP's level of expertise in neurology and epilepsy.

If so, contrary to our study, all the studies mentioned above show: (1) that videos analyzed by experts serve as an excellent means of distinguishing between ES and PNES and (2) that the ability to differentiate between ES and PNES depends on the level of expertise of the viewers.

Therefore, if we could enhance the ability of viewers to distinguish between ES and PNES to the level of experts, we could significantly advance the proper and early diagnosis and treatment of these patients even before they reach epileptologists.

The GDI principle, developed by our epilepsy team, is based on the dynamism of movements observed during seizures. This study sought to validate the GDI principle and evaluate its effectiveness in enabling both trained and untrained personnel to accurately diagnose seizures using video footage.

Our results demonstrated that GDI training significantly improved the accuracy of diagnosis among observers. The mean scores of observers who received GDI training (Groups 2 and 3) were significantly higher than those who did not (Group 1), indicating that the training effectively enhanced their ability to distinguish between PNES and ES. Specifically, the mean score of Group 2 was 75.55 compared to 55.23 in Group 1 (*p* = 0.0000001), and Group 3 improved from 53 to 78 after training (*p* = 0.000001).

Moreover, the sensitivity and specificity of the GDI principle in diagnosing PNES were 75.73% and 77.67%, respectively. These findings suggest that the GDI tool is a reliable method for seizure classification, even when used by personnel with varying levels of medical experience.

The simplicity and brevity of the GDI training (approximately 5 min) make it a practical tool for clinical settings. This is particularly important for first responders and medical staff in emergency departments, who often encounter patients with seizures, and must make rapid diagnostic decisions. The ability to accurately identify PNES and ES without the need for extensive training or specialized equipment can improve patient outcomes by ensuring that individuals receive appropriate treatment promptly.

Several similar studies have explored different methods to differentiate between PNES and ES, emphasizing the importance, and challenge of accurate diagnosis. For instance, Reuber et al. (2002) conducted a study that highlighted the limitations of clinical history, and physical examination in distinguishing PNES from ES, underscoring the need for more reliable diagnostic tools (Reuber et al. 2002). In addition, a study by Hubsch et al. (2011) examined the use of automated analysis of seizure semiology, finding that certain movement patterns, and durations could help differentiate PNES from ES, albeit with the need for sophisticated software, and analysis tools (Hubsch et al. 2011).

Furthermore, the work by Avbersek and Sisodiya (2010) reviewed the effectiveness of various diagnostic tools, including VEM, and emphasized the potential of video‐based assessments in improving diagnostic accuracy (Avbersek and Sisodiya 2010). Their findings align with our study, suggesting that video analysis, when combined with specific training, can significantly enhance the differentiation between PNES and ES.

Video teaching interventions have been shown to improve the ability of medical students to diagnose PNES (Seneviratne et al. 2014.). In that study, the students had a 1‐h interactive lecture on diagnosing and classifying ES and PNES. The study results showed improvement, from a diagnosis that is no better than chance to poor diagnostic ability, with the decay of knowledge over time.

While the initial feasibility of the GDI principle shows promise, there are limitations to our study. The GDI principle is applicable solely to motor seizures. The sample size was relatively small, and the observers were from a single healthcare campus, which may limit the generalizability of the results. In addition, the GDI principle's absolute validity has not been fully established, and further research is needed to confirm its effectiveness in larger, more diverse populations.

It's important to emphasize that while we recognize the significance of the GDI principle, we do not recommend that clinicians rely on it exclusively for diagnosing PNES or ES. Instead, we advocate for a comprehensive approach that incorporates various diagnostic tools, as discussed earlier. However, for the purposes of this study, we focused solely on testing the isolated effect of the GDI.

Future studies should aim to replicate these findings in different clinical settings and with a broader range of participants. It would also be beneficial to explore the long‐term impact of GDI training on clinical practice and patient outcomes. In addition, integrating GDI into telemedicine platforms could further enhance its utility, particularly in remote or resource‐limited settings.

## 5 Conclusion

This study demonstrates the potential of the GDI principle as an effective tool for differentiating between PNES and ES using video footage. The significant improvement in diagnostic accuracy among observers following GDI training highlights its value as a practical, accessible method for seizure classification. Continued research and broader implementation of the GDI tool could significantly enhance the diagnosis and management of seizures, ultimately improving patient care and outcomes.

## Author Contributions

**Linor Avraham**: investigation, project administration. **Ronen Spierer**: validation, formal analysis. **Raz Winer**: validation, data curation. **Noam Bosak**: data curation, validation, visualization. **Danielle Wasserman**: validation. **Moshe Herskovitz**: conceptualization, writing–original draft, writing–review and editing, supervision.

## Conflicts of Interest

The authors declare no conflicts of interest.

### Peer Review

The peer review history for this article is available at https://publons.com/publon/10.1002/brb3.70130.

## Supporting information



Video 1: The global dynamic impression principle is explained in its first half. The second half of the video includes examples of seizures and the use of the principle. The first seizure with movements lacking a specific pattern—an unpredictable “dance”—is epileptic. The second example is a tonic–clonic seizure, characterized by a crescendo‐decrescendo pattern. Watch as each movement changes direction over time. The third example is a psychogenic nonepileptic seizure, featuring repeated short episodes of hand jerks. Lastly, the fourth case, where a patient moves multiple body parts with constant direction and rhythm. The convulsion begins in a sudden burst and ends abruptly.

## Data Availability

Anonymized data will be shared by request from any qualified investigator.
